# Obsessive compulsive disorder as a presenting symptom of COVID-19: A case-series from Iran

**DOI:** 10.22088/cjim.13.0.259

**Published:** 2022

**Authors:** Mahsa Owji, Abdorreza Naser Moghadasi, Faezeh Gholamian, Seyed Yaser Mousavi

**Affiliations:** 1 Multiple Sclerosis Research Center, Neuroscience institute, Tehran University of Medical Sciences, Tehran, Iran; 2 Department of Psychiatry, Roozbeh Psychiatric Hospital, Tehran University of Medical Sciences, Tehran, Iran; 3 Department of Psychiatry, Children’s Medical center, Tehran University of Medical Sciences, Tehran, Iran

**Keywords:** COVID-19, Obsessive compulsive disorder, Neuropsychiatric presentation

## Abstract

**Background:**

Coronavirus disease 2019 (COVID-19) involves different organs and clinical presentations varying from respiratory symptoms to gastrointestinal symptoms and neurological symptoms. Another group of symptoms are psychiatric symptoms and according to a study, 53.8% of participants reported various degrees of these symptoms.

**Case Presentation:**

In this study, we presented six cases with sudden onset OCD or exacerbation of the previously controlled OCD concomitant with COVID-19 symptoms.

**Result:**

Obsessive compulsive symptoms can be the presenting manifestation of COVID-19.

**Conclusion:**

The neuropsychiatric manifestations may be resulted from central nervous system (CNS) infiltration, and dysregulation of inflammatory factors autoimmune responses.

Ccoronavirus disease 2019 (COVID-19) currently is a great pandemic started from Wuhan, China. It involves different organs and clinical presentations varying from respiratory symptoms including dry cough, dyspnea, respiratory distress, and nasal congestion to gastrointestinal symptoms including nausea, vomiting, and diarrhea and neurological symptoms such as headache, encephalopathy, and cerebrovascular disease (1). Moreover, the disease can also be asymptomatic (2). Notably, children may also become infected by the disease and the symptoms can be fever, cough, respiratory distress, and gastrointestinal symptoms (3). Another group of symptoms are psychiatric symptoms and according to a study, 53.8% of participants reported various degrees of these symptoms (4). Patients may suffer from anxiety disorders, mood disorders, sleep disorders, and may also have suicidal ideas (2). The neuropsychiatric manifestations may be resulted from Central Nervous System (CNS) infiltration, and dysregulation of inflammatory factors autoimmune responses (2). In this regard, obsessive compulsive disorder (OCD) is characterized by some ideas, impulses, thoughts or urges coming to mind unwantedly, which are also called "obsessions”, repetitive behaviors or drives done in response to obsessions that are called "compulsions" (5). Accordingly, some studies suggested that immune responses may play a role in pathophysiology of OCD in adults (6). In children with a sudden onset OCD, there exists a term called PANS *(pediatric acute-onset neuropsychiatric syndrome) due to *doubt about the etiology (7). However, an immunological cause has been considered. In this study, we presented six cases with sudden onset OCD or exacerbation of the previously controlled OCD concomitant with COVID-19 symptoms.

## Case presentation

Mr. A was a 16-year-old adolescent who came to the emergency ward with fever, cough, and a sudden onset of obsessional symptoms such as frequent washing of mouth to prevent transfer of infection, verbal checking the parents on the occurrence of the disease, and frequent washing the hands for a long time. He was admitted to the hospital after performing laboratory evaluations with the diagnosis of COVID-19. According to mild symptoms and not having respiratory distress, he underwent supportive treatment, and due to the severity of obsessional symptoms, psychiatric consultation was requested. 10 mg capsule of fluoxetine per day was prescribed to him. Afterwards, the patient was discharged from the emergency ward 2 days later and was then quarantined at home for 14 days. After 1 month of starting fluoxetine, he went to the psychiatry clinic. The severity of obsessional symptoms was relatively declined, so he was recommended to continue pharmacotherapy with fluoxetine at a dose of 20 mg per day.

Mr. B, a 42-year-old man referred to us with fever; respiratory distress; cough; sleep disturbance as 2 hours delay in the beginning of sleep; and a sudden onset of obsessional symptoms such as worriedness about hygiene of food, frequent checking of the doors and windows’ closeness at home, and irritability, so he came to the emergency ward and was admitted to the hospital at the intensive care unit (ICU) because of respiratory distress, with the diagnosis of COVID-19. On the third day of admission, respiratory distress aggravated, at the same time gastrointestinal bleeding occurred and liver enzymes have elevated. Unfortunately, the patient passed away. Notably, brain magnetic resonance imaging (MRI) was reported as normal.

Mr. C, a 23-year-old man referred to the hospital with fever, cough, headache, and sudden exacerbation of obsessional symptoms such as frequent hand washing and lengthy shower for about 1.5-2 hours. After performing the evaluation, he was diagnosed with COVID-19 and underwent supportive care. The patient had a history of obsession of doubt and orderliness since he was 17 years old and was under the treatment with sertraline at a dose of 150 mg per day. Notably, before getting infected by the disease, the symptoms had been significantly controlled. The patient regularly consumed the medications up to the time of referring to the hospital. Psychiatric consultation was performed and according to the severity of obsessional symptoms, the dose of sertraline was increased to 200 mg per day. Moreover, due to obsessional thoughts and irritability, 0.25 mg oral haloperidol for night consumption was prescribed. Because his physical condition was stable, after performing the required evaluations, he was discharged and quarantined at home. However, one month later, with a relative improvement of obsessional symptoms, he was referred to the clinic, haloperidol was discontinued, and he was recommended to maintain sertraline.

Mr. D, a 13-year-old adolescent referred to the emergency ward with fever, cough, sore throat, diarrhea, irritability, and a sudden onset of obsessional symptoms such as orderliness, lengthy showering, and obsessional thoughts like sexual obsessions. He was admitted to the hospital with the diagnosis of COVID-19. Due to electrolyte disturbance and the elevated liver enzymes, he was transferred to ICU. At the third day of admission, he developed tonic clonic seizure. Treatment of obsessional symptoms was postponed up to the time of the stabilization of physical condition due to the severity of physical condition. Notably, brain MRI was reported as normal.

Ms. E, a 9-year-old girl referred to the emergency ward with fever; sore throat; headache, restlessness; anxiety symptoms; and a sudden onset of obsessional symptoms like worries on being infected with the disease and severe restlessness when attending the public, repeated hand washing, severe sensitivity to the orderliness of her room, preventing the entrance of others to her room, and not eating meals with her family and eating alone at her room. After performing the evaluation, COVID-19 was diagnosed and due to the stability of physical condition, home quarantine was recommended. Because of having anxiety and obsessional symptoms, psychiatric consultation was requested and oral fluoxetine 5mg per day was started for her. In one month follow-up, obsession symptoms have relatively improved and cognitive behavioral therapy (CBT) was also initiated. A significant improvement was seen in the obsession symptoms of the child after 2 months.

Ms. F was a 14-year-old adolescent who referred to the clinic with fever, rhinorrhea, and a sudden onset of obsessional symptoms such as doubt in saying prayers correctly and repeating its words to come to a correct pronunciation, not leaving home since the beginning of the coronavirus pandemic, repeated mouth and nose washing, and religious obsessional thoughts. Fluoxetine capsule 10 mg per day was initiated for her and due to having suspicion on being infected with COVID-19, she was referred for further evaluations. With the diagnosis of COVID-19 and according to her favorable physical condition, home quarantine was recommended. In a one-month follow-up, no considerable changes were seen in the severity of obsession symptoms. Therefore, fluoxetine dose was increased to 20 mg per day and 0.25 mg oral haloperidol was added to the treatment. In the next visit that was one month later, a relative improvement was observed in the severity of obsession symptoms. The diagnosis of these patients was according to DSM-5 criterion (8). The patients’ laboratory findings were shown in table 1.

**Table 1:** Patients’ laboratory findings

**Test (Unit)**	**A**	**B**	**C**	**D**	**E**	**F**
SARS-CoV2 IgM	Positive	positive	positive	positive	positive	Positive
SARS-CoV2 IgG	Negative	negative	negative	negative	negative	Negative
RT-PCR	Positive	positive	positive	positive	positive	Positive
WBC (µL)	13.7*$10^{3}$	9.0*$10^{3}$	5.2*$10^{3}$	11.6*$10^{3}$	8.9*$10^{3}$	13.6*$10^{3}$
Neutrophil (%)	78.0	82.1	43.5	71.2	76.0	95.5
Lymphocyte (%)	22.0	16.0	46.6	19.7	21.3	3.05
Hemoglobin (gr/dl)	12.8	11.4	12.0	11.1	13.1	12.3
PLT	292*$10^{3}$	101*$10^{3}$	142*$10^{3}$	168*$10^{3}$	282*$10^{3}$	157*$10^{3}$
ESR (mm/h)	21	72	36	28	14	26
CRP (mg/l)	++	+++	+	++	++	++
AST (U/L)	49	31	19	23	48	29
ALT (U/L)	41	24	17	29	37	23
Urea (mg/dl)	53	96	71	43	45	25
Creatinine (mg/dl)	1.1	4.4	1.7	1.2	1.1	1.3
PT (Sec)	13	13.5	-	14	-	-
PTT (Sec)	28	33	-	30	-	-
INR	1.1	1.2	-	1.2	-	-
Albumin Serum (G/dl)	3.1	3.3	3.7	3.1	3.5	3.1
Sodium (mEq/L)	143	142	133	140	135	135
Potassium (mEq/L)	4.8	4.3	3.9	4.8	4.0	4.1
Magnesium (mg/dl)	1.99	2.23	2.00	2.30	2.21	1.63
Calcium (mg/dl)	7.8	7.9	8.0	9.4	8.8	8.2
BS (mg/dl)	95	150	113	78	112	95

Abbreviations: WBC, White Blood Cell; PLT, Platelet; ESR, Erythrocyte Sedimentation Rate ; CRP, C-Reactive Protein ; AST, Aspartate Transaminase; ALT, Alanine Transaminase; Blood Urea Nitrogen; PT, Prothrombin Time ; PTT, Partial Thromboplastin Time ; INR, International Normalized Ratio ; BS, Blood Suger

## Discussion

COVID-19 is a disease with various presentations resulting from the involvement of different body organs, and respiratory and gastrointestinal symptoms are more prevalent (9). However, psychological symptoms, except some case reports (10-12), have not been recognized as primary symptoms and as common presentations of COVID-19. Common symptoms among patients included in our study, similar to many other reports, were fever, respiratory and gastrointestinal symptoms. However, a difference was observed that was in all of them, a sudden onset or sudden exacerbation of previous obsessive-compulsive symptoms was concurrent with the beginning of other clinical symptoms and these symptoms were among the prominent symptoms of the disease presentation. This can be considered as an evidence for neuropsychiatric involvement following infection with COVID-19. Such symptoms were previously considered in ageless children as PANS syndrome following viral infections, and involvement of adolescents and adults was very rare (13). But herein, most of the patients were adolescents or adults, and this age difference in the incidence of symptoms similar to PANS is notable. 

PANS is recognized as an immunological disorder which occurs in response to any kind of infection (14). In a similar way, it seems that there exists a kind of immunological correlation between these new presenting symptoms and COVID-19. There are different reports in literature indicating autoimmune disorders following COVID-19 (15-17). Some of them have psychological presentations and OCD is not an exception. 

Another important point in this study was the presence of similar psychological symptoms in patients with different severities of COVID-19, which informs the need for paying more attention to psychological symptoms. It seems that, psychological and neuropsychiatric presentations are along with a sudden onset, exacerbation or uncommon presentations, as well as fever or some respiratory or gastrointestinal symptoms. Even mild ones at any age, should raise the suspicion of COVID-19 in clinicians, and consequently necessary diagnostic evaluations should be performed. It is obvious that lack of attention and incorrect diagnosis can have dangerous consequences to the patient as well as the possibility of spreading and transmitting the infection to others. 

This study has important limitations. Imaging or metabolic characteristics of these patients were not evaluated. These evaluations could explain the main mechanism of developing OCD after catching COVID-19 in these patients. In addition, Yale Brown test was not used to evaluate changes in disease severity or response to treatment. These points should be noted in similar case reports.
